# Crystallization and preliminary X-ray diffraction analyses of several forms of the CfaB major subunit of enterotoxigenic *Escherichia coli* CFA/I fimbriae

**DOI:** 10.1107/S1744309109001584

**Published:** 2009-02-14

**Authors:** Yong-Fu Li, Steven Poole, Fatima Rasulova, Annette L. McVeigh, Stephen J. Savarino, Di Xia

**Affiliations:** a Laboratory of Cell Biology, Center for Cancer Research, National Cancer Institute, NIH, Bethesda, MD 20892-4256, USA; b Enteric Diseases Department, Infectious Diseases Directorate, Naval Medical Research Center, Silver Spring, MD 20910-7500, USA; c Department of Pediatrics, Uniformed Services University of the Health Sciences, Bethesda, MD 20814-4799, USA

**Keywords:** colonization factor antigen I fimbriae, CfaB subunit, enterotoxigenic *Escherichia coli*

## Abstract

Enterotoxigenic *Escherichia coli* (ETEC), a major global cause of diarrhea, initiates the pathogenic process *via* fimbriae-mediated attachment to the small intestinal epithelium. A common prototypic ETEC fimbria, colo­nization factor antigen I (CFA/I), consists of a tip-localized minor adhesive subunit CfaE and the stalk-forming major subunit CfaB, both of which are necessary for fimbrial assembly. To elucidate the structure of CFA/I at atomic resolution, three recombinant proteins were generated consisting of fusions of the minor and major subunits (CfaEB) and of two (CfaBB) and three (CfaBBB) repeats of the major subunit. Crystals of CfaEB diffracted X-rays to 2.1 Å resolution and displayed the symmetry of space group *P*2_1_. CfaBB exhibited a crystal diffraction limit of 2.3 Å resolution and had the symmetry of space group *P*2_1_2_1_2. CfaBBB crystallized in the monoclinic space group *C*2 and diffracted X-­rays to 2.3 Å resolution. These structures were determined using the molecular-replacement method.

## Abstract 2

Three fusion proteins were generated in order to resolve the atomic structure of the CFA/I fimbriae of enterotoxigenic *E. coli*. CfaEB is a fusion of the minor and major CFA/I subunits, while CfaBB and CfaBBB are tandem fusions of two and three repeats, respectively, of the major subunit. Each protein was crystallized and the crystal structures of each of these fusions were determined successively by the molecular-replacement method using the CfaE crystal structure as an initial phasing model.

## 1. Introduction

Although it has been almost 40 years since it was first implicated as a prevalent cause of travelers’ diarrhea and as a leading bacterial cause of diarrhea morbidity and mortality in young children in developing countries (Rowe *et al.*, 1970 ▶; Black *et al.*, 1981 ▶), human-specific enterotoxigenic *Escherichia coli* (ETEC) has until recently largely escaped structural examination of its adhesive machinery. Central to the pathogenesis of ETEC-induced secretory diarrhea is the ability of the organism to adhere to the small intestinal mucosa *via* adhesive fimbriae or colonization factors (Turner *et al.*, 2006 ▶). Although nearly two dozen such colonization factors have been described, little is known about the molecular details of their structure and function (Gaastra & Svennerholm, 1996 ▶; Steinsland *et al.*, 2003 ▶).

Colonization factor antigen I (CFA/I) was the first such adhesive fimbria to be discovered and is the archetype of eight class 5 ETEC fimbriae (Evans *et al.*, 1978 ▶; Anantha *et al.*, 2004 ▶). Of the other seven class 5 fimbriae, CS1 fimbriae have been extensively studied, particularly their biogenesis and regulation (Sakellaris *et al.*, 1996 ▶, 1999 ▶; Munson *et al.*, 2002 ▶). Like CS1, CFA/I is encoded by a four-gene operon and is assembled by the alternate chaperone pathway, which has been distinguished from the classic chaperone–usher pathway that guides the assembly of class I pili such as type 1 and P pili (Soto & Hultgren, 1999 ▶; Anantha *et al.*, 2004 ▶). CFA/I fimbriae are heteropolymeric structures that are composed of a tip-localized minor adhesive subunit (CfaE) subjoined to a homopolymeric tract of >1000 CfaB major subunits (Sakellaris & Scott, 1998 ▶; Poole *et al.*, 2007 ▶; Li *et al.*, 2007 ▶; Mu *et al.*, 2008 ▶). Bioassembly is orchestrated by a periplasmic chaperone (CfaA), which promotes proper subunit folding and delivery to an outer membrane usher protein (CfaC), which extrudes the subunits in an ordered fashion to form a regular helical superstructure (Sakellaris & Scott, 1998 ▶; Anantha *et al.*, 2004 ▶; Mu *et al.*, 2008 ▶). The donor-strand complementation and exchange mechanism, first discovered in structural investigations of type 1 and P pili (Sauer *et al.*, 1999 ▶; Choudhury *et al.*, 1999 ▶), also appears to be a hallmark of class 5 fimbrial biogenesis (Poole *et al.*, 2007 ▶; Li *et al.*, 2007 ▶).

Recent structural studies have begun to elucidate the molecular details of CFA/I fimbriae. We have reported the crystal structure of the CfaE tip adhesin, which shows similarities to the two-domain structures of certain other Gram-negative adhesins, including FimH from type 1 fimbriae and PapG from P pili (Li *et al.*, 2007 ▶). A three-dimensional helical reconstruction of CFA/I fimbriae has also been reported based on transmission electron microscopy of a negatively stained CFA/I specimen, revealing a right-handed helix for the CfaB filament with weak inter-coil interactions (Mu *et al.*, 2008 ▶).

Building upon this structural framework will require atomic level details of the structure of the major subunit CfaB. In approaching this aim, we have drawn upon the difficulties and successes encountered in atomic structure determination of major subunits of class I pili as well as nonclassical filamentous structures of the chaperone–usher pathway. Only recently has the first major subunit of a classical helically coiled pilus structure been determined: that of PapA, which required the introduction of several mutations and cocrystallization with its chaperone (Verger *et al.*, 2007 ▶). In contrast, the structures of a number of major subunits of nonclassical fibrillar or afimbrial sheath structures have been solved using various approaches (Zavialov *et al.*, 2003 ▶; Pettigrew *et al.*, 2004 ▶; Anderson *et al.*, 2004 ▶; Van Molle *et al.*, 2007 ▶). After unsuccessful attempts to crystallize a unitary *in cis* donor-strand complemented form of CfaB, we adopted a novel strategy to tandemly fuse two or more CFA/I subunits in order to emulate the native noncovalent linkages formed by donor-strand exchange. Here, we report the engineering of three different recombinant fusion proteins each containing one or more CfaB units with a C-terminal extension comprising the donor β-strand of CfaB to achieve protein stability. Each of these proteins, consisting of in-tandem arrangements of minor–major, major–major and major–major–major subunits, were purified in soluble form and crystallized. The structure solutions of these three fusions are expected to provide the structural basis for dissecting the function of CFA/I fimbriae at the submolecular level.

## 2. Materials and methods

### 2.1. Construction of expression vectors for recombinant CfaEB, CfaBB and CfaBBB fusion proteins

#### 2.1.1. Construction of pET24-*2lnkdsc*_*19*_ *cfaEB(his)*_*6*_

The *cfaB* gene was amplified by PCR using the primers *cfaB* (reverse), 5′-GTG­GTGGTGGTGGTGCTCGAGGGATCCCAAAGTCATTACAAG­AGA-3′, and *cfaB* (forward), 5′-GTAGAGAAAAATATTACTGTAACAGC-3′, using pNTP513 as template (Hibberd *et al.*, 1991 ▶). The resulting product was inserted at the 5′-end of *cfaE* in pET24-*dsc*_*19*_ *cfaE(his)*_*6*_ (Poole *et al.*, 2007 ▶; Li *et al.*, 2006 ▶) by site-directed mutagenesis (QuikChange Kit, Stratagene, La Jolla, California, USA) to form the intermediate construct pET24-*lnkcfaEB(his)*_*6*_. The coding sequence for a short linker (DNKQ) followed by the N-­terminal donor β-strand (19 residues) of CfaB (‘DNKQ-dsc19’) was amplified by PCR from pET24-*dsc*_*19*_ *cfaE(his)*_*6*_ using primers containing *Bam*HI and *Xho*I sites (forward, 5′-CGCCGC**GGATCC**GACAATAAACAAGTAGAGAAAAATATT-3′; reverse, 5′-CCGCCG**CTCGAG**TTGCAAAAGATCAATCACAGGATC-3′). Digestion of pET24-*lnkcfaEB(his)*_*6*_ and the ‘DNKQ-dsc19’ PCR product with *Bam*HI and *Xho*I and subsequent ligation yielded the final plasmid construct pET24-*2lnk* *dsc*_*19*_ *cfaEB(his)*_*6*_. This plas­mid, which contains an LEHH­HHHH tag at the C-terminus for ease of purification, was transformed into *E. coli* BL21(DE3) for expression.

#### 2.1.2. Construction of pET24-*2lnkdsc*_*15*_ *cfaBB(his)*_*6*_

The *cfaB* gene was amplified by PCR from the wild-type ETEC strain H10407 (ATCC) with the primers 5′-A**CATATG**ATTGATCTTTTGCAAGCTGATGGC-3′ (forward) and 5′-CTCGAG**AATTGCAGGATCA** **ACACTAGCTGTTA**CAGTAATATTTTTCTCTACCTGTTTGTTATCGGATCCCAAAGTCATTACAAGAGATACTAC-3′ (reverse); the latter contains the coding sequence for ‘DNKQ-dsc15’. The *cfaB* fragment was then cloned into pET24a(+) pre-digested with *Nde*I and *Xho*I. Subsequently, the *cfaB* gene was PCR-amplified again with two pairs of primers [the *Nde*I and *Sac*I pair, 5′-A**CATATG**ATTGATCTTTTGCAAGCTGATGGC-3′ (forward) and 5′-AGAGCTCAATT­GCAGGATCAACACTAGCTGTTA-3′ (reverse), and the *Sac*I and *Xho*I pair 5′-AGAGCTCTTGCAAGCTGATGGCAATGCTCTG­CCA-3′ (forward) and 5′-AAGCTTAATTGCAGGATCAACACTA­GCTGTTA-3′ (reverse)], which were cloned into the TOPO cloning vector pCRXL-TOPO separately and transformed into OneShot Top10F competent cells. Each *cfaB* gene was confirmed by DNA sequencing and the respective plasmids were digested with either *Nde*I and *Sac*I or *Sac*I and *Xho*I. DNA fragments were recovered after separation by agarose-gel electrophoresis and ligated into similarly digested vector pET24a(+). The desired plasmids with two tandem *cfaB* gene segments were identified by restriction analysis and confirmed by DNA sequencing. The pET24-*2lnkdsc*_*15*_ *cfaBB(his)*_*6*_ plasmid, which contains an LEHHHHHH tag at the C-­terminus for ease of purification, was transformed into *E. coli* BL21(DE3) for expression.

#### 2.1.3. Construction of pET24-*3lnkdsc*_*15*_ *cfaBBB(his)*_*6*_

Similarly, pET24-*3lnkdsc*_*15*_ *cfaBBB(His)*_*6*_ was constructed with three copies of *cfaB* amplified using three sets of primers [the *Nde*I and *Sac*I pair, 5′-­TAACAGCTAGTGTTGATCCTGCAATTTGAAAGCTT-3′ (for­ward) and 5′-AGAGCTCAATTGCAGGATCAACACTAGCTGTT­A-3′ (reverse), the *Sac*I and *Hin*dIII pair, 5′-AGAGCTCTTGCAAGCTGATGGCAATGCTCTGCCA-3′ (forward) and 5′-AAGCT­TAATTGCAGGATCAACACTAGCTGTTA-3′ (reverse), and the *Hin*dIII and *Xho*I pair, 5′-AAGCTTTTGCAAGCTGATGGCAAT­GCTCTGCCA-3′ (forward) and 5′-CTCGAGAATTGCAGGATCA­ACACTAGCTGTTACAGTAATATTTTTCTCTACCTGTTTGTTA­TCGGATCCCAAAGTCATTACAAGAGATACTAC-3′ (reverse)], which were subsequently cloned into the pCRXL-TOPO plasmid and confirmed by DNA sequencing. The three *cfaB* genes were released with respective restriction enzymes and ligated to pET24a(+) pre-digested by *Nde*I and *Xho*I to yield pET24-*3lnkdsc*_*15*_ *CfaBBB(His)*_*6*_. This plasmid also contains an LEHHHHHH tag at the C-terminus for ease of purification.

### 2.2. Expression and purification of CfaEB, CfaBB and CfaBBB

#### 2.2.1. Purification of dscCfaEB(His)_6_

Cultures of BL21(DE3)/pET24-*2lnkdsc_*19*_dsc*_*19*_ *cfaEB(his)*_*6*_ were grown at 305 K in the alternative protein source Super Broth (Difco, Detroit, Michigan, USA) with 50 µg ml^−1^ kanamycin to late logarithmic phase and induced with 1 m*M* IPTG for 3 h. Harvested cell pellets were resuspended in 1:4(*w*:*v*) buffer *A* (20 m*M* phosphate, 500 m*M* NaCl, 50 m*M* imidazole pH 7.4) and subjected to disruption by microfluidization (Model M110-Y Apparatus, Microfluidic Corp., Newton, Massachusetts, USA). The lysate was centrifuged at 17 000*g* for 45 min at 277 K. The supernatant was loaded onto a HisTrap FF column (GE Healthcare, Piscataway, New Jersey, USA) equilibrated with buffer *A*. Protein was eluted with a gradient to 300 m*M* imidazole over 20 column volumes (CVs). Fractions containing the protein of interest were resolved by SDS–PAGE and detected by Western blotting using anti-dscCfaE antibodies (Poole *et al.*, 2007 ▶). These fractions were pooled and diluted tenfold with buffer *B* (25 m*M* MES pH 6.0) before loading onto a HiTrap SP column (GE Healthcare, Piscataway, New Jersey, USA) equilibrated in buffer *B*. Protein was eluted using a gradient to 500 m*M* NaCl over 20 CVs. Fractions containing dsc_19_CfaEB(His)_6_ (hereafter called CfaEB) were pooled, concentrated with an Amicon Ultra-15 centrifugal filter (Millipore, Billerica, Massachusetts, USA) and applied onto a Superdex 75 10/300 GL column equilibrated with phosphate-buffered saline pH 6.7. Fractions containing CfaEB were pooled and concentrated to ∼10 mg ml^−1^. The purity of the final pooled sample was determined by densitometric analysis of an SDS–PAGE gel. The protein concentration was determined using the BCA assay (Pierce, Rockford, Illinois, USA) and its identity was confirmed by N-terminal sequence analysis and Western blotting using anti-dscCfaE and anti-dscCfaB antibodies (Poole *et al.*, 2007 ▶).

#### 2.2.2. Purification of dscCfaBB(His)_6_ and dscCfaBBB(His)_6_

The procedures used for the purification of dscCfaBB and dscCfaBBB were identical. Specifically, BL21(DE3) strain harboring either pET24-*2lnkdsc*_*15*_ *cfaBB(his)*_*6*_ or pET24-*3lnkdsc*_*15*_ *cfaBBB(his)*_*6*_ was grown at 310 K in Super Broth supplemented with 50 µg l^−1^ kanamycin and induced with 1 m*M* IPTG. Cells were washed, suspended in phosphate-buffered saline (PBS) containing a protease-inhibitor cocktail (Sigma, St Louis, Missouri, USA), and disrupted by two passes through a French Press operated at 10.3 MPa. After centrifugation, the supernatant was applied onto Ni–NTA resin and eluted with an automated program controlled by the ÄKTA FPLC system (GE Healthcare) in a buffer containing 20 m*M* Tris–HCl pH 8.0, 0.5 *M* NaCl and a varying imidazole concentration from 10 to 500 m*M*. The dsc_15_CfaBB(His)_6_ or dsc_15_CfaBBB(His)_6_ (hereafter called CfaBB or CfaBBB, respectively) fractions were pooled and ammonium sulfate (AS) was added to achieve 40% saturation before application onto a Phenyl-Sepharose column pre-equilibrated with 40% saturated AS in 20 m*M* Tris–HCl pH 7.5. The elution gradient was 40–0% AS in the same buffer. Purified CfaBB or CfaBBB fractions were pooled and dialyzed against a buffer containing 20 m*M* Tris–HCl pH 7.5 with 100 m*M* NaCl. To determine the apparent molecular weight, purified CfaBB/CfaBBB was analyzed on a Superdex 200 size-exclusion column operated in a buffer consisting of 20 m*M* Tris–HCl pH 7.5, 200 m*M* NaCl.

### 2.3. Crystallization, diffraction data collection and reduction

CfaEB, CfaBB and CfaBBB were crystallized using the vapor-diffusion method at 288 K. Typically, initial crystallization screening was performed robotically with a Mosquito automated solution dispenser (TTP LabTech) coupled with commercially available high-throughput screening kits (Hampton Research and Molecular Dimensions) in a hanging-drop format. Each droplet was a mixture of 300 nl protein and 300 nl reservoir solution and a volume of 50 µl reservoir solution was employed. Conditions for initial hits were repeated and confirmed with solutions prepared in-house. The initial conditions were identified as D1, D6 and D11 of MemStart MemSys HT96 from Molecular Dimensions for CfaEB, while that for CfaBB was found to be G12 of IndexHT from Hampton Research and those for CfaBBB were A10, B8, D6 and F1 of Crystal Screen HT from Hampton Research. For optimization, additive screening kits from commercial screens (Hampton Research) were used in a high-throughput setting. Productive crystallization followed optimization by setting up droplets containing equal volumes of protein and reservoir solution at 2–3 µl and placing each droplet over 0.5 ml reservoir solution. Crystal clusters with estimated sizes up to 1 mm could be obtained within 7–10 days at 288 K.

Crystals were tested for diffraction quality and for cryoprotection in-house with a Rigaku RU-H3R X-ray generator and a MAR345 imaging-plate scanner. The X-ray diffraction data sets reported in this study were collected at 100 K using either a MAR300 CCD or a MAR225 CCD detector on the SER-CAT beamline of the Advanced Photon Source (APS), Argonne National Laboratory (ANL). The raw diffraction data were processed using the program *HKL*-2000 (Otwinowski & Minor, 1997 ▶). Statistics indicating the quality of the diffraction data sets are given in Table 1 ▶.

## 3. Results and discussion

CFA/I fimbriae contain a single copy of CfaE, a tip-localized adhesive subunit, and >1000 copies of CfaB, the stalk-forming major subunit. Both are necessary for fimbrial assembly. We have previously reported the crystal structure of an *in cis* donor-strand complemented form of CfaE (Li *et al.*, 2006 ▶, 2007 ▶; Poole *et al.*, 2007 ▶). However, solution of the crystal structure of CfaB is a prerequisite for a complete and more detailed understanding of the general function of CFA/I at submolecular resolution.

### 3.1. Strategy in making CfaB fusion proteins

In the reported crystal structure of CfaE, the donor-strand com­plementation principle was employed to engineer an *in cis* donor-strand complemented CfaE (dscCfaE) by covalently attaching a peptide fragment (donor strand) from the N-terminus of CfaB to the C-terminal end of CfaE, thereby filling in the hydrophobic groove of CfaE for the missing G-strand to complete the IgG fold. We sought to use the same approach for the structure solution of the major subunit CfaB. An expression vector for the production of donor-strand complemented CfaB (dscCfaB) was constructed and protein was purified, but the purified dscCfaB never crystallized owing to its extraordinary solubility in solution even at a protein concentration as high as 80 mg ml^−1^ (data not shown). A different approach was then devised by extending the donor strand in the dscCfaE construct into the main body of CfaB to create the fusion protein CfaEB; the extended CfaB domain was again donor-strand complemented *in cis*. The resulting fusion protein is better suited to crystallization and for solving the crystallographic phase problem since the structure of CfaE is already known (see below).

An added benefit of the CfaEB fusion is that it may provide the geometric relation between the two pilin subunits in the native pilus. Similarly, structure determinations for the fusion proteins of two or three major pilin subunits connected in tandem, CfaBB and CfaBBB, are essential for constructing an atomic model of the CFA/I pilus.

### 3.2. Protein purification and crystallization

**Figure 1:**
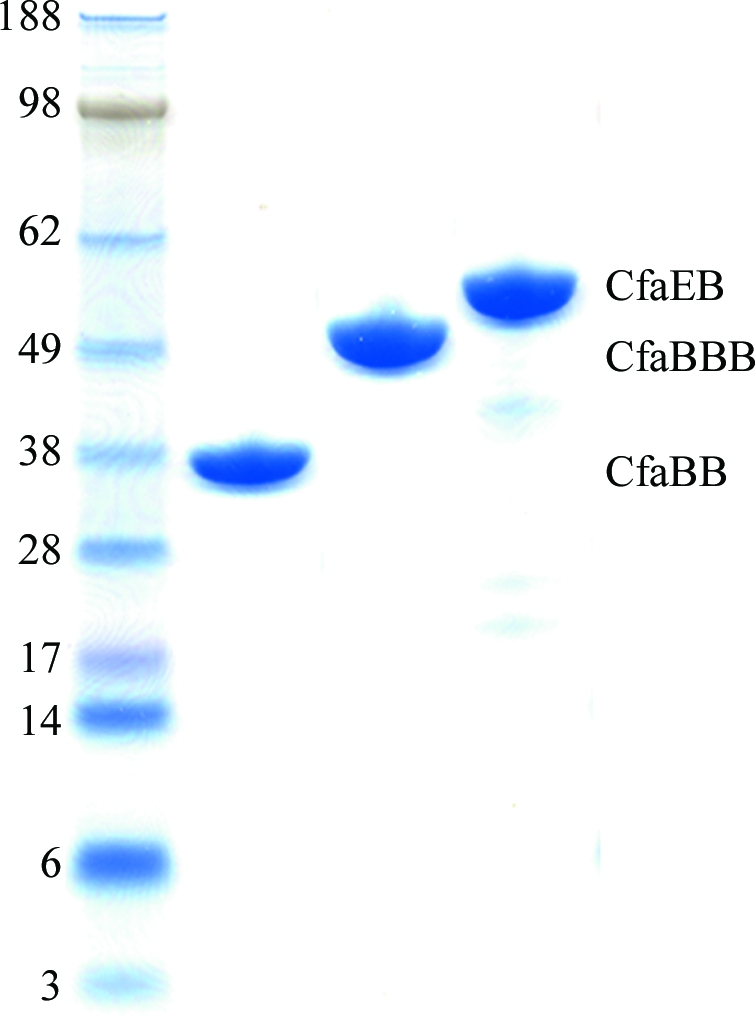
SDS–PAGE of purified CfaEB, CfaBB and CfaBBB.

The *pET24-2lnkdsc*_*19*_ *cfaEB(his)*_*6*_, *pET24-2lnkdsc*_*15*_ *cfaBB(his)*_*6*_ and *pET24-3lnkdsc*_*15*_ *cfaBBB(his)*_*6 *_plasmids for expression of the donor-strand complemented CfaEB heterodimeric, CfaBB homodimeric and CfaBBB homotrimeric fusions were constructed by insertion into a pET24a(+) plasmid with genes coding for covalent minor–major, major–major and major–major–major pilin fusions, respectively. Short DNA sequences coding for DNKQ-dsc19 (Poole *et al.*, 2007 ▶) and DNKQ-dsc15 were incorporated in two positions for CfaEB and CfaBB and in three positions for CfaBBB, between the two genes and after the last CfaB, to complete the donor-strand complementation. A hexahistidine affinity tag is present at the C-­terminus in all constructs After transformation into *E. coli* strain BL21(DE3), protein overexpression was obtained for all constructs upon IPTG induction. While CfaEB was purified by sequential nickel-affinity column and ion-exchange chromatography, CfaBB and CfaBBB were purified by nickel-affinity chromatography followed by hydrophobic chromatography (Fig. 1 ▶).

**Figure 2:**
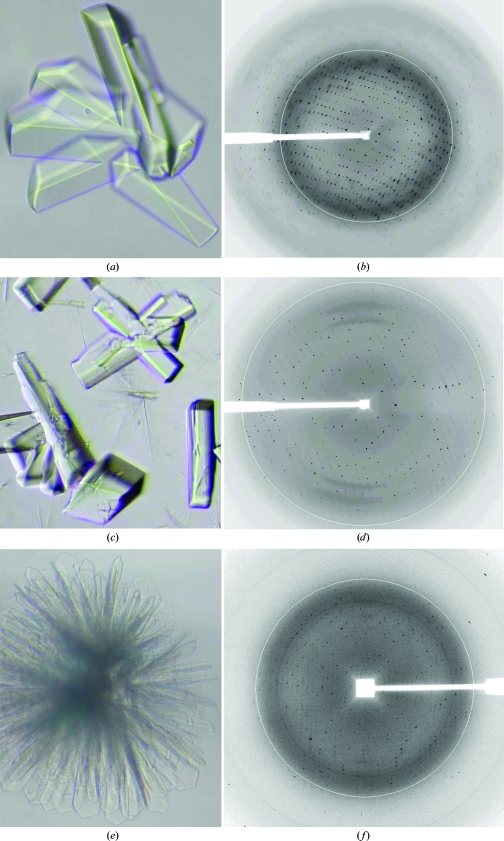
Crystals and X-ray diffraction patterns for CfaEB, CfaBB and CfaBBB. (*a*) Typical crystals of CfaEB. (*b*) X-ray diffraction pattern of a CfaEB crystal. (*c*) Crystals of CfaBB. (*d*) Diffraction image of a CfaBB crystal. (*e*) A clustered crystal of CfaBBB. (*f*) Diffraction pattern of a CfaBBB crystal severed from the crystal shown in (*e*). The circles in (*b*), (*d*) and (*f*) are 3 Å resolution markers.

Each purified protein was analyzed by size-exclusion chromatography to ensure monodispersity and concentrated to approximately 10 mg ml^−1^ before crystallization experiments. The CfaEB protein was solubilized in a buffer containing 20 m*M* MES pH 6.0 plus 100 m*M* NaCl. The final crystallization condition for CfaEB was a mixture in a hanging-drop setup of 1 µl protein solution with 1 µl well solution consisting of 10–11% PEG 8000, 200 m*M* ammonium sulfate, 100 m*M* citrate pH 4.0. For CfaBB crystallization, 10 mg ml^−1^ protein in a buffer containing 20 m*M* Tris–HCl pH 7.5 in the presence of 200 m*M* NaCl was mixed in a 1:1 ratio with a well solution containing 30% PEG 8000 and 200 m*M* ammonium sulfate. Similarly, the CfaBBB protein (10 mg ml^−1^ in 20 m*M* Tris–HCl pH 7.5, 100 m*M* NaCl) was crystallized by mixing it in a 1:1 ratio with 22% PEG 4000, 100 m*M* ammonium sulfate, 100 m*M* sodium citrate pH 3.5, 1% ethylene glycol, 2% PEG 400, 1% 2-propanol, 10 m*M* MgCl_2_ and 0.3% 1,2,3-heptanetriol. This condition was obtained after optimization by pH and additive screening. Crystals of CfaEB often grew in clusters with well defined morphology (Fig. 2 ▶ *a*), whereas those of CfaBB and CfaBBB exhibited rod-like shapes with rough surfaces and also formed clusters (Figs. 2 ▶ *c* and 2 ▶ *e*).

### 3.3. Cryoprotection and initial X-ray diffraction analysis

We found that an additional 10% PEG 400 was sufficient for cryoprotection of all crystals during freezing and diffraction data collection. Crystals of CfaEB were well shaped and often formed clusters (Fig. 2 ▶ *a*). Crystals (0.1 × 0.1 × 0.2 mm) in a cluster were separated prior to X-ray diffraction experiments and gave a diffraction limit beyond 2 Å resolution (Fig. 2 ▶ *b*). The crystals belonged to a monoclinic space group, with unit-cell parameters *a* = 67.14, *b* = 45.16, *c* = 128.32 Å, β = 97.31°. The merged data set was 92.0% complete to 2.10 Å resolution, with an *R*_merge_ of 6.2% and a mean *I*/σ(*I*) of 7.0 (Table 1 ▶). A screw axis must be present, as noted from systematic absences for 0*k*0 (*k* = 2*n* + 1) reflections, permitting the assignment of space group *P*2_1_. The Matthews coefficient (*V*_M_) was calculated as 3.2 Å^3^ Da^−1^, assuming the presence of one molecule of CfaEB per crystallographic asymmetric unit, indicating a solvent content of about 62% (Matthews, 1968 ▶).

Crystals of CfaBB were considerably more radiation-sensitive than those of CfaEB. Fortunately, these crystals belonged to a higher symmetry orthorhombic space group (Fig. 2 ▶ *d*) and the time required to com­plete a data-collection run was further reduced by short exposure times. Although the diffraction limits for CfaBB crystals were similar to those of CfaEB, the merged data set was 96.5% complete only to 2.25 Å resolution, with an *R*_merge_ of 9.5% and an average *I*/σ(*I*) of 5.6 (Table 1 ▶). Systematic absences indicated that these crystals possessed the symmetry of space group *P*2_1_2_1_2. The calculated *V*_M_ value was 2.5 Å^3^ Da^−1^, assuming the presence of two CfaBB molecules in the asymmetric unit, with a solvent content of about 51% (Matthews, 1968 ▶).

More so than CfaBB crystals, CfaBBB crystals tended to cluster (Fig. 2 ▶ *e*). Crystals used for diffraction data collection had to be severed with a knife from the tips of the cluster. These crystals were cryoprotected for data collection and diffracted X-rays to better than 2 Å resolution using synchrotron radiation (Fig. 2 ▶ *f*). CfaBBB crystals had the symmetry of space group *C*2 and unit-cell parameters *a* = 127.53, *b* = 44.81, *c* = 98.11 Å, β = 125.41°. A data set with 93.5% completeness was obtained at 2.10 Å resolution (Table 1 ▶) with a merging *R* factor of 0.079. A *V*_M_ value of 3.2 Å^3^ Da^−1^ was obtained based on the presence of a single CfaBBB molecule in the asymmetric unit.

### 3.4. Phase determination

Because the structure of a donor-strand complemented adhesive subunit CfaE from CFA/I fimbriae (PDB code 1hb0) has recently been reported (Li *et al.*, 2006 ▶, 2007 ▶), the crystallographic phase problem could be solved for the CfaEB fusion crystal by the molecular-replacement (MR) method, obviating the need to obtain heavy-metal or selenomethionine derivatives. A clear solution with a *Z* score of approximately 15 was obtained with the MR program *Phaser* (Storoni *et al.*, 2004 ▶). Initial refinement with *REFMAC* (Murshudov *et al.*, 1997 ▶) in the *CCP*4 program suite (Collaborative Computational Project, Number 4, 1994 ▶) using the *Phaser*-generated CfaE coordinates gave rise to an *R* factor and *R*_free_ of 0.372 and 0.394, respectively, and produced clear additional electron density corresponding to the CfaB domain in the fusion, permitting model building of the major pilin subunit. With the unrefined coordinates for the major pilin subunit CfaB, MR with *Phaser* was carried out on the CfaBB data set; four solutions were obtained, representing two CfaBB fusion molecules per asymmetric unit. The *R* factor and *R*_free_ for the first cycle of refinement with *REFMAC*5 were 0.303 and 0.326, respectively. The CfaBBB data set was similarly phased using the coordinates of the CfaB subunit from the CfaEB structure. When all three CfaB subunits had been identified and put into refinement in *REFMAC*5 in the CfaBBB structure, the *R* factor and *R*_free_ for the initial cycle were 0.235 and 0.341, respectively. Model building, refinement and structure description of the CfaEB, CfaBB and CfaBBB fusions will be reported separately.

## Figures and Tables

**Table 1:** Characterization of CfaEB, CfaBB and CfaBBB crystals and their diffraction statistics Values in parentheses are for the outer resolution shell.

	CfaEB	CfaBB	CfaBBB
Wavelength (Å)	0.7500	1.0000	0.7500
Beamline	22-ID, APS	22-ID, APS	22-ID, APS
Exposure time (s)	5	2	4.5
Resolution (Å)	50–2.10 (2.18–2.10)	50–2.25 (2.33–2.25)	50–2.10 (2.18–2.10)
Space group	*P*2_1_	*P*2_1_2_1_2	*C*2
Unit-cell parameters			
*a* (Å)	67.14,	75.21	127.53
*b* (Å)	45.16	134.82	44.81
*c* (Å)	128.32	65.07	98.11
α (°)	90	90	90
β (°)	97.31	90	125.41
γ (°)	90	90	90
No. of observations	290802	184126	124305
No. of unique reflections	44915	32280	25005
Mosaicity (°)	0.642	0.227	0.375
*R*_merge_†	0.062 (0.229)	0.095 (0.388)	0.079 (0.401)
Completeness (%)	92.0 (75.5)	96.5 (83.4)	93.5 (71.9)
Average *I*/σ(*I*)	23.0 (7.1)	15.4 (2.4)	17.3 (3.4)
Redundancy	7.0	5.6	5.0

† *R*_merge_ is defined as 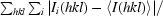 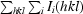, where *I_i_*(*hkl*) is the intensity for the *i*th observation of a reflection with Miller indices *hkl* and 〈*I*(*hkl*)〉 is the mean intensity for all measured values of *I*(*hkl*) and its Friedel pair.
